# A multicenter phase II study of biweekly capecitabine in combination with oxaliplatin as first-line chemotherapy in patients with locally advanced or metastatic gastric cancer

**DOI:** 10.1007/s00280-014-2407-y

**Published:** 2014-02-17

**Authors:** Yee Chao, Jan-Sing Hsieh, Hsien-Tang Yeh, Yu-Chieh Su, Cheng-Chung Wu, Jen-Shi Chen, Cheng-Jeng Tai, Li-Yuan Bai, Kun-Huei Yeh, Wu-Chou Su, Chung-Pin Li

**Affiliations:** 1 Department of Oncology Medicine, Taipei Veterans General Hospital, Taipei, Taiwan; 2 National Yang-Ming University School of Medicine, Taipei, Taiwan; 3 Department of Surgery, Kaohsiung Medical University Hospital, Kaohsiung, Taiwan; 4 Department of Surgery, Faculty of Medicine, College of Medicine, Kaohsiung Medical University, Kaohsiung, Taiwan; 5 Department of Surgery, Lotung Poh-Ai Hospital, Yilan County, Taiwan; 6 Division of Hematology–Oncology, Department of Internal Medicine, Buddhist Dalin Tzu Chi General Hospital, Chiayi, Taiwan; 7 Division of General Surgery, Department of Surgery, Taichung Veterans General Hospital, Taichung, Taiwan; 8 Division of Hematology-Oncology, Department of Internal Medicine, Chang Gung Memorial Hospital and Chang Gung University, Taoyuan County, Taiwan; 9 Division of Hematology and Oncology, Department of Internal Medicine, Taipei Medical University Hospital, Taichung, Taiwan; 10 Department of Internal Medicine, School of Medicine, College of Medicine, Taipei Medical University, Taichung, Taiwan; 11 Division of Hematology and Oncology, Department of Internal Medicine, China Medical University Hospital and School of Medicine, College of Medicine, China Medical University, Taichung, Taiwan; 12 Department of Oncology, National Taiwan University Hospital and National Taiwan University College of Medicine, Taipei, Taiwan; 13 Division of Hematology-Oncology, Department of Internal Medicine and Graduate Institute of Clinical Medicine, National Cheng Kung University Hospital, College of Medicine, National Cheng Kung University, Tainan, Taiwan; 14 Division of Gastroenterology, Department of Medicine, Taipei Veterans General Hospital, No. 201, Sec. 2, Shih-Pai Road, Taipei, 11217 Taiwan

**Keywords:** Gastric cancer, Phase II study, Biweekly XELOX, Capecitabine, Oxaliplatin

## Abstract

**Purpose:**

We evaluated the safety and efficacy of biweekly capecitabine in combination with oxaliplatin in previously untreated patients with locally advanced or metastatic gastric cancer.

**Methods:**

Patients received oral capecitabine 1,000 mg/m^2^ twice daily on days 1–10 plus oxaliplatin 85 mg/m^2^ as a 2-h intravenous infusion on day 1, every 2 weeks (XELOX). The primary endpoint was overall response rate. Secondary endpoints included progression-free survival, overall survival, and toxicity.

**Results:**

From March 2007 to October 2010, 46 patients were enrolled in this phase II study. The median age was 64 years (range 32–85). A total of 391 (median 7.5, range 1–29) cycles were delivered. Among the 41 patients evaluable for tumor response, 9 showed partial response and 25 had stable disease. The overall response rates of the evaluable and intent-to-treat (ITT) populations were 22 % (95 % CI 10–42 %) and 20 % (95 % CI 9–34 %), respectively. In the ITT analysis, the progression-free survival and overall survival were 5.6 months (95 % CI 4.1–6.3 months) and 8.0 months (95 % CI 6.3–10.1 months), respectively. The most common hematological toxicities were thrombocytopenia (35 %) and leucopenia (34 %), whereas the most common non-hematological toxicities were neuropathy (35 %), fatigue (33 %), diarrhea (27 %), vomiting (26 %), and hand-foot syndrome (25 %). Major grade 3–4 toxicities were anemia (11 %), diarrhea (9 %), and hand-foot syndrome (7 %). No patient died of treatment-related toxicities.

**Conclusions:**

Although the biweekly XELOX regimen failed its primary response rate endpoint, it showed modest efficacy and an acceptable safety profile in the treatment of advanced gastric cancer.

## Introduction

Gastric cancer is the fourth most common and the second leading cause of cancer deaths in the world. According to global estimates, gastric cancer was newly diagnosed in approximately 989,600 people and caused approximately 738,000 deaths in 2008, with the highest rates occurring in Eastern Asia [1]. Although early diagnosis of gastric cancer has improved, most patients still show locally advanced or metastatic disease upon diagnosis. Even if surgery is performed with a curative intent, 60 % of patients with resectable tumors will ultimately relapse [2].

It has been demonstrated that combination chemotherapy regimens in advanced gastric cancer improve overall survival and progression-free survival of patients and maintain quality of life [3]. 5-Fluorouracil (5-FU) in combination with cisplatin is a generally accepted standard treatment option for advanced gastric cancer patients [4–8]. The combination regimen of 5-FU and cisplatin achieves overall response rates of 20–51 %, median progression-free survival of 3.3–6.5 months, and median overall survival of 7–11 months [4–8]. Nevertheless, the required weekly 24-h intravenous infusion of 5-FU is inconvenient, cumbersome, and associated with the risk of infection and thrombosis.

Capecitabine, a novel oral fluoropyrimidine designed to mimic the continuous infusion of 5-FU, is administered orally. After oral administration, it is efficiently absorbed from the gastrointestinal tract and metabolized primarily in the liver. Capecitabine is then sequentially converted to the cytotoxic 5-FU moiety in tumor tissues by the enzyme thymidine phosphorylase, which shows significantly higher activities in tumors than in normal tissues [9, 10]. In several phase II studies, capecitabine has shown good response rates in advanced gastric cancer patients when provided as monotherapy or in combination with cisplatin, achieving response rates of 19–34 and 55 %, respectively [11–14]. Oral fluoropyrimidine administration can eliminate the inconvenience and the risks (i.e., venous thrombosis and sepsis) associated with the infusion of 5-FU. Furthermore, capecitabine showed non-inferior effects compared with 5-FU in gastric cancer patients [15].

Oxaliplatin, initially developed to treat colorectal cancer, is an alkylating agent that forms adducts between two adjacent guanines or a guanine and an adenine residue, leading to the inhibition of DNA replication [16]. Compared with cisplatin, oxaliplatin appears to have a more favorable tolerability profile. In particular, renal toxicity and ototoxicity are not associated with oxaliplatin, but are commonly encountered during cisplatin therapy [17]. The dose-limiting toxicity of oxaliplatin is a cumulative sensory peripheral neuropathy. In randomized phase III trials, oxaliplatin was non-inferior to cisplatin in the treatment of esophagogastric cancer [18, 19].

Triweekly oxaliplatin plus oral capecitabine has become a new standard for the treatment of advanced gastric cancer [20–25], achieving response rates of 42–63 % in published phase II studies [20–24]. These results warrant further investigation into this drug combination in patients with gastrointestinal malignancies. A biweekly regimen of the same chemotherapy combination has been shown to be effective in metastatic colorectal cancer [26], but biweekly data are currently not available for advanced gastric cancer. The aim of this study was to demonstrate the efficacy and safety of a biweekly regimen of capecitabine in combination with oxaliplatin in previously untreated patients with locally advanced or metastatic gastric cancer.

## Patients and methods

### Study design

This is a multicenter study to investigate the safety and efficacy profiles of biweekly capecitabine (Xeloda^®^) in combination with oxaliplatin (Eloxatin^®^) as first-line therapy in patients with locally advanced or metastatic gastric cancer. The primary objective was to investigate the objective response rate of capecitabine plus oxaliplatin treatment in previously untreated locally advanced or metastatic gastric cancer patients. Secondary objectives included progression-free survival, duration of response, overall survival (OS), and safety profiles. The study protocol was approved by the medical ethics committees of all participating centers and was registered as ClinicalTrial.gov (NCT00436241). Signed informed consents were obtained from all patients.

### Patients

Inclusion criteria for this study were as follows: histologically confirmed gastric adenocarcinoma with unresectable locally advanced or metastatic disease; at least one, non-irradiated, measurable lesion according to the Response Evaluation Criteria in Solid Tumors (RECIST) [27]; 18 years of age or older; calculated creatinine clearance ≥50 ml/min using the Cockroft–Gault formula; and Eastern Cooperative Oncology Group (ECOG) performance status ≤1.

Patients who fell under any of the following criteria were excluded from the study: previous cytotoxic chemotherapy (except given as adjuvant or neoadjuvant treatment completed at least 6 months prior to enrollment); organ allografts; clinically significant cardiac disease or myocardial infarction within the last 12 months; evidence of CNS metastases; history of another malignancy within the last five years except for cured basal cell carcinoma of the skin or cured carcinoma in situ of the uterine cervix; radiotherapy within 4 weeks of treatment start; major surgery within 4 weeks of the start of the treatment, without complete recovery; serious uncontrolled intercurrent infections; lack of physical integrity of the upper gastrointestinal tract or malabsorption syndrome; abnormal audiogram or auditory abnormality; significant or uncontrolled gastrointestinal bleeding; the following laboratory values: neutrophils ≤1.5 × 10^9^/L; platelet count <100 × 10^9^/L; serum bilirubin ≥1.5 × upper normal limit; alanine aminotransferase or aspartate aminotransferase >2.5 × upper normal limit or >5 × upper normal limit in the case of liver metastases; alkaline phosphatase >2.5 × upper normal limit or >5 × upper normal limit in the case of liver metastases or >10 × upper normal limit in the case of bone disease.

### Treatment schedule

Capecitabine (1,000 mg/m^2^ twice daily, days 1–10, followed by four days of rest period) plus oxaliplatin (85 mg/m^2^ as a 2-h intravenous infusion on day 1) were administered in two-week cycles. Protocol treatment was discontinued for patients with clearly documented progressive disease (PD) at any time due to insufficient therapeutic response. Patients who tolerated treatment and showed either complete response (CR), partial response (PR), or stable disease (SD) continued to be treated and followed until disease progression.

### Dose modification for adverse events

The intensity of clinical adverse events was graded according to the National Cancer Institute (NCI) Common Terminology Criteria for Adverse Events v3.0 (CTCAE). Dose modifications were based on hematological and non-hematological toxicities.

For hematological toxicities, dose modifications were based on hematological parameters at the start of a treatment cycle. Administration of capecitabine was interrupted during a treatment cycle if a grade 3 or 4 hematological toxicity developed. The next treatment cycle could only start if hematological toxicity has recovered to grade ≤1. No dose reduction or interruption was required for anemia (non-hemolytic) as it could be satisfactorily managed by transfusions.

For non-hematological toxicities of capecitabine, if a grade 2 or 3 adverse event occurred, capecitabine was interrupted immediately; if a grade 4 non-hematological toxicity occurred, treatment was discontinued unless the investigator considered it to be in the best interest of the patient to continue at 50 % of the original dose, once toxicity has resolved to grade 0–1. The next treatment cycle could only start if non-hematological toxicity has recovered to grade ≤1. Capecitabine dose was reduced by 25 % for patients experiencing a second occurrence of a given grade 2 or any grade 3 event. The capecitabine dose was reduced by 50 % for patients who experienced a third occurrence of a given grade 2 or a second occurrence of a given grade 3 event. If the same toxicity occurred for a fourth time at grade 2 or a third time at grade 3, treatment was discontinued.

For grade <3 non-hematological toxicities of oxaliplatin, management was symptomatic, if possible. For grade ≥3 non-hematological adverse events, oxaliplatin was withheld for a maximum of 4 weeks until toxicities were resolved. After patients recovered from toxicity grade 3–2 or less, the dose of oxaliplatin was reduced to 65 mg/m^2^ in subsequent cycles. In case of no resolution to grade 2 or less after a maximum of 4 weeks from the planned date of the next cycle, oxaliplatin treatment was discontinued. In case of grade 4 toxicity, patients were removed from oxaliplatin treatment and followed until the resolution of the adverse event.

### Evaluation of efficacy and toxicities

Evaluations, including the patient’s medical history, physical examination, complete blood count, blood chemistry, abdominal–pelvic computed tomography (CT) scan, and chest X-ray, were performed before chemotherapy. After starting the protocol treatment, blood chemistry and complete blood count were assessed prior to the start of each cycle. Tumor measurements were carried out after every 3 cycles of treatment or when progression was suspected. Confirmation of overall response (complete or partial response), when applicable, was done at a minimum of 4 weeks after the first response had been recorded. Tumor response was evaluated according to the Response Evaluation Criteria in Solid Tumors (RECIST) [27]. Toxicities were recorded according to the National Cancer Institute Common Terminology Criteria grading system version 3, starting at the time of study entry and ending 28 days after the last dose of medication was administered.

### Statistical methods

Simon’s two-stage optimal design was applied to calculate the sample size, assuming that the minimum response rate of capecitabine plus oxaliplatin in the study was 29 % and the expected overall response rate was at least 50 %. Under *α* = 0.05 and 80 % power, the estimated total evaluable patient number was 38 to detect more than 15 patients having partial response. Considering a 10 % drop-out rate, the estimated enrolled patient number was 42. Progression-free survival was measured as the duration from the date of starting protocol treatment to the date of first recording disease progression or the date of death, whichever occurred first. Overall survival was measured as the duration from the date of starting protocol treatment to the date of death. Duration of response was measured from the first date when measurement criteria were met for CR/PR to the first date when recurrent or progressive disease or death was objectively documented. Survival was estimated by the Kaplan–Meier analysis. All statistics were two-sided and performed using SAS software (version 9.2, SAS Inc., Cary, NC, USA).

## Results

### Patient’s characteristics

Between March 2007 and November 2008, forty-six patients (ITT population) from ten medical centers in Taiwan were enrolled in this study. The clinical and pathologic characteristics of patients are listed in Table 1.

**Table 1:** Clinicopathologic features of the patients

	Patient number (%)
Total patients	46
Age (years), median (range)	64 (32–85)
Sex, male/female	31/15
Race	
Oriental	46 (100)
ECOG performance	
0	25 (54)
1	21 (46)
Disease status	
Locally advanced	3 (7)
Recurrence/metastasis	43 (93)
Differentiation	
Well differentiated	1 (2)
Moderately differentiated	11 (24)
Poorly differentiated	21 (46)
Not assessable	13 (28)
Metastatic site^a^	
Liver	23 (50)
Lymph nodes	20 (43)
Peritoneum	13 (28)
Lung	6 (13)
Bone	5 (11)
Pleura	3 (7)
Soft tissue	2 (4)
Others	13 (28)

^a^Patients could have more than one site of metastasis

### Efficacy

The tumor response rates are shown in Table 2. Five patients could not be evaluated for response due to failure to return for tumor measurements. Among the 41 evaluable patients, the best tumor response was partial response (PR) in 9 patients, whereas 25 patients showed stable disease and 7 patients progressive disease. The overall response rates of the evaluable and intent-to-treat (ITT) populations were 22 % (95 % CI 10–42 %) and 20 % (95 % CI 9–34 %), respectively, whereas the disease control rates in the corresponding populations were 83 % (95 % CI 68–93 %) and 74 % (95 % CI 59–86 %), respectively. Median time to tumor response was 1.4 months (range 1.2–2.8 months). The median duration of response was 5.1 months (95 % CI 2.8–9.7 months). Of the 46 patients, 26 received second-line and subsequent therapies, with a total of 47 additional regimens received. Among them, six patients received monotherapy (2 UFUR, 1 capecitabine, 1 everolimus, 1 irinotecan, and 1 paclitaxel), 9 patients received cisplatin plus fluoropyrimidine-based therapy, 4 patients received anthracycline-based therapy with various combinations of 5-FU, cisplatin, and cyclophosphamide, 4 patients received taxane-based therapy, 3 patients received oxaliplatin-based therapy, 4 patients received irinotecan-based therapy, and 2 patients received 5-FU plus cyclophosphamide or cetuximab. Three additional patients also received mainly fluoropyrimidine-based subsequent therapy.

**Table 2:** Treatment response (*n* = 46)

Responses	Number of patients	% of patients
Overall response rate (CR + PR)	9	20
Complete response	0	0
Partial response	9	20
Stable disease	25	54
Progressive disease	7	15
Not assessable	5	11

*CR* complete response, *PR* partial response

**Fig. 1:**
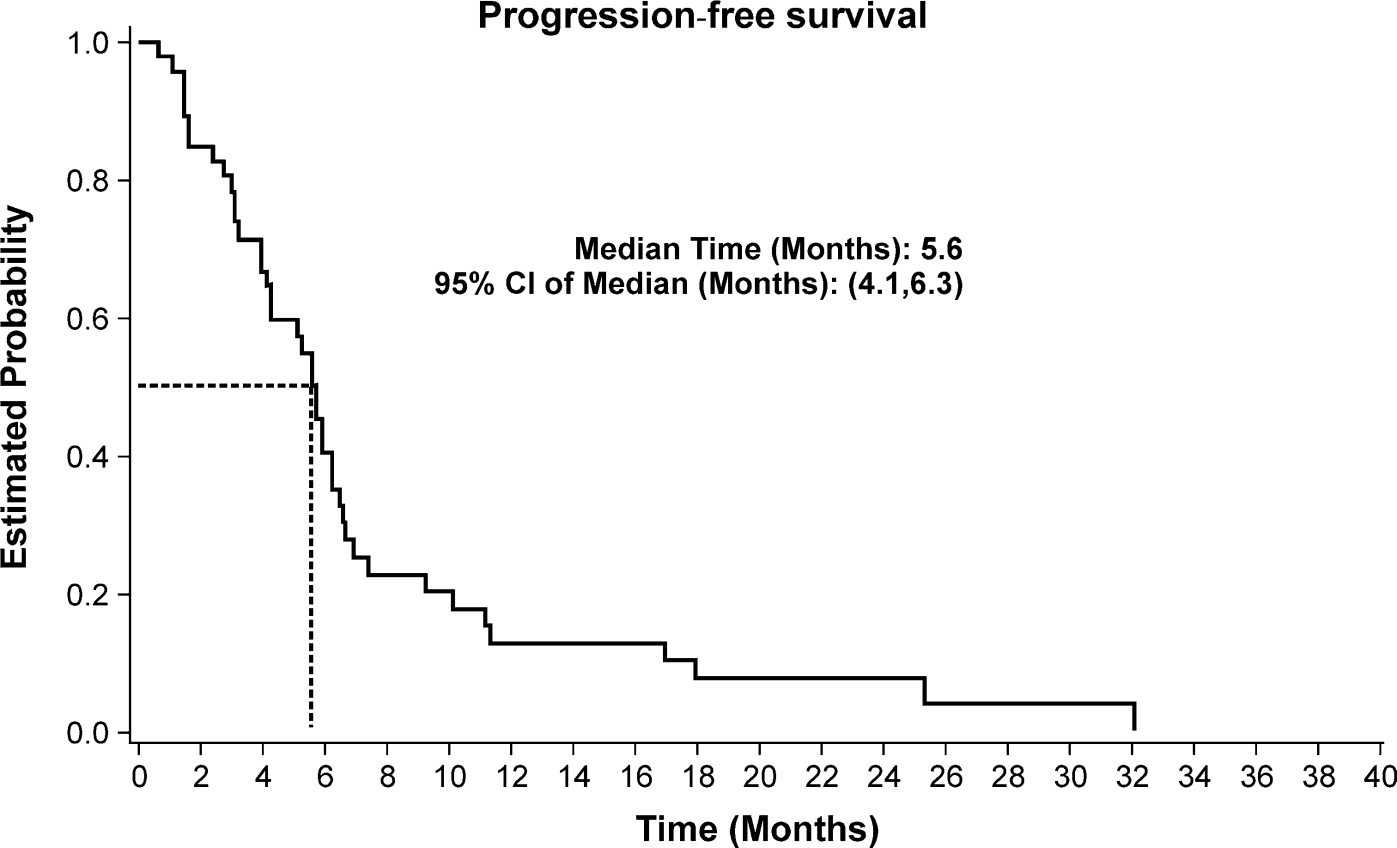
Progression-free survival of the 46 patients

**Fig. 2:**
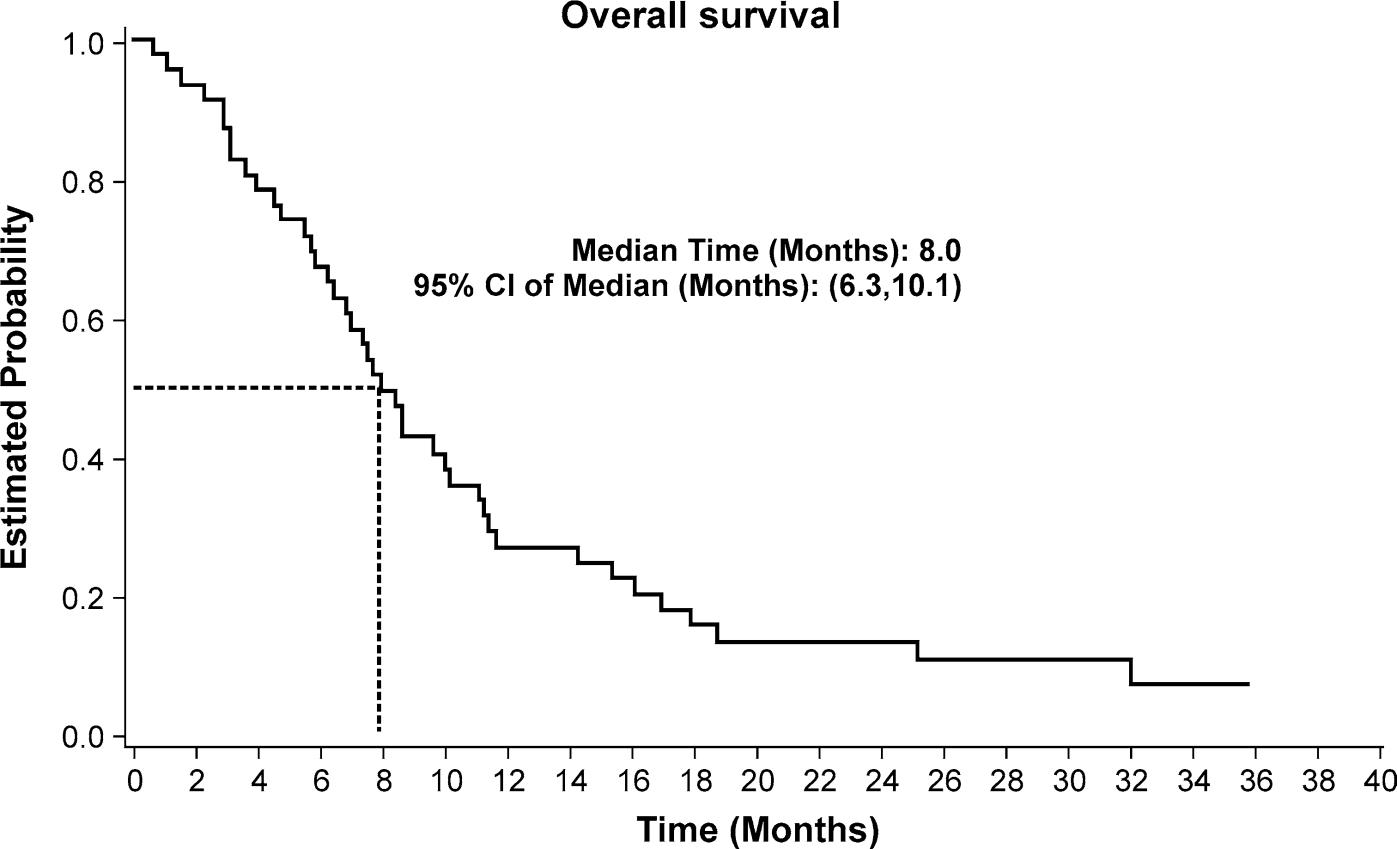
Overall survival of the 46 patients

Median follow-up time was 7.8 months (range 0.7–35.9 months). Median progression-free survival and overall survival were 5.6 months (95 % CI 4.1–6.3 months) and 8.0 months (95 % CI 6.3–10.1 months), respectively. The Kaplan–Meier estimated progression-free survival and overall survival curves are shown in Figs. 1 and 2, respectively. Overall survival at 1 year was 26.9 % and at 2 years was 13.4 %.

### Safety

A total of 391 (median 7.5, range 1–29) cycles of chemotherapy were given. The median relative dose intensity was 88.5 % (range 51.4–103.2 %) for capecitabine and 99.3 % (range 43.6–103.9 %) for oxaliplatin. In total, 82.6 % of the patients received more than 80 % of the intended dose of capecitabine, and 95.7 % received more than 80 % of the intended dose of oxaliplatin.

All patients were evaluated for toxicities (Table 3). The most common hematological toxicity was thrombocytopenia (33 %), but without grade 3 or 4 events. Grade 3 leucopenia was observed in 2 % of the patients. Grade 3–4 anemia developed in five patients (11 %). The most common non-hematological toxicities were neuropathy (35 %), fatigue (31 %), diarrhea (26 %), vomiting (26 %), and hand-foot syndrome (22 %). Major grade 3 toxicities were diarrhea (9 %) and hand-foot syndrome (7 %). Treatment was delayed in 82 cycles (21 %), and dose modifications were implemented in 13 cycles (3 %). The most frequent cause of treatment delays was thrombocytopenia (31/82; 38 %), and the major reason for dose modification was hand-foot syndrome (6/13; 46 %). There were no treatment-related deaths.

**Table 3:** Toxicities associated with the biweekly capecitabine plus oxaliplatin regimen

Toxicity	Grade			
	1	2	3	4
Hematological				
Leucopenia	15	17	2	0
Neutropenia	9	21	0	0
Thrombocytopenia	20	13	0	0
Anemia	4	9	9	2
Gastrointestinal				
Mucositis	11	4	2	0
Anorexia	20	4	0	0
Nausea	9	4	2	0
Vomiting	9	11	4	2
Diarrhea	11	7	9	0
Constipation	9	7	0	0
Abdominal pain/distension	9	9	2	0
Weight loss	9	11	4	0
Neurological				
Neuropathy	26	7	2	0
Insomnia	13	2	0	0
Dizziness	9	0	0	0
Others				
Hand-foot syndrome	11	4	7	0
Fatigue	28	2	0	0
Fever	4	2	2	0
Pitting edema	11	4	2	0
Hypertension	2	2	2	0

All numbers are percentages of the 46 patients

## Discussion

A combination chemotherapy regimen of 5-FU plus cisplatin has been widely used for the treatment of advanced gastric cancer patients [4–8]. However, this regimen is inconvenient, cumbersome, and associated with the risk of infection and thrombosis due to the continuous 5-FU infusion. In addition, cisplatin has a poor tolerability, due mostly to its renal toxicity.

Triweekly oxaliplatin plus oral capecitabine has been used for treating advanced gastric cancer, with response rates of 42–63 % and overall survival of 10.0–11.9 months [20–24]. In the treatment of metastatic colorectal cancer, biweekly oxaliplatin plus oral capecitabine has been shown to be as active as oxaliplatin plus intravenous 5-FU/leucovorin, with similar quality of life for patients [26]. However, the efficacy and toxicities of biweekly oxaliplatin plus oral capecitabine in treating advanced gastric cancer have not been investigated so far. This is the first phase II trial of using biweekly oxaliplatin plus oral capecitabine in advanced gastric cancer.

In a multicenter open-label phase II study, oral capecitabine has been shown to be an active and well-tolerated treatment in gastric cancer patients [13]. In a phase III trial of capecitabine in advanced gastric cancer, it has shown a non-inferior effect and similar safety profile to 5-FU [15]. In two published phase III studies, oxaliplatin was non-inferior to cisplatin and resulted in reduced toxicity compared with cisplatin in the treatment of esophagogastric cancer patients [18, 19]. Based on these published articles, capecitabine and oxaliplatin are considered as effective as 5-FU and cisplatin, respectively. In addition, administration of capecitabine is more convenient for patients than infusion of 5-FU, whereas oxaliplatin is more tolerable than cisplatin. Therefore, capecitabine and oxaliplatin are able to replace 5-FU and cisplatin, respectively.

The aim of this phase II study was to demonstrate the efficacy and safety of biweekly capecitabine in combination with oxaliplatin in previously untreated patients with locally advanced or metastatic gastric cancer. In our study, the overall response rate of ITT patients was 20 %. Median progression-free survival was 5.6 months, and median overall survival was 8.0 months. Although this biweekly XELOX study failed to meet its primary response rate endpoint, it still showed modest efficacy in the treatment of advanced gastric cancer.

An important conclusion from our phase II multicenter study was that the biweekly combination regimen of capecitabine and oxaliplatin had a good safety profile. Only 2 patients withdrew from the study because of treatment-related toxicities, and there were no treatment-related deaths. Grade 3 and 4 hematological and non-hematological adverse events were infrequent, and all toxicities were generally of mild to moderate intensity. The most common adverse events were thrombocytopenia (33 %), neuropathy (35 %), leucopenia (35 %), neutropenia (31 %), fatigue (31 %), diarrhea (26 %), vomiting (26 %), anemia (24 %), and hand-foot syndrome (22 %). The safety profile reported in our trial compared favorably with other phase II clinical studies with triweekly combination regimens of capecitabine/oxaliplatin [20–24]. Neuropathy, the major distressing toxicity of oxaliplatin, occurred in 35 % of our patients, which is relatively low compared to percentages reported in other studies (22–70 %) [20–24]. Hand-foot syndrome was also infrequent relative to other triweekly capecitabine/oxaliplatin studies (22 vs. 20–39 %) [20–24]. Neutropenia also occurred in a lower percentage of patients in this study than in other reports (31 vs. 35–56 %) [20–24].

In conclusion, the combination of biweekly capecitabine and oxaliplatin shows modest activity and an acceptable safety profile. The therapeutic index of the current regimen, which can be helpful to advanced gastric cancer patients, should be further explored.
